# A comparison of amplification methods to detect Avian Influenza viruses in California wetlands targeted via remote sensing of waterfowl

**DOI:** 10.1111/tbed.13612

**Published:** 2020-06-27

**Authors:** Madeline M. McCuen, Maurice E. Pitesky, Jeffrey J. Buler, Sarai Acosta, Alexander H. Wilcox, Ronald F. Bond, Samuel L. Díaz‐Muñoz

**Affiliations:** ^1^ Department of Population Health and Reproduction School of Veterinary Medicine‐Cooperative Extension University of California Davis CA USA; ^2^ Department of Entomology and Wildlife Ecology University of Delaware Newark DE USA; ^3^ Department of Microbiology and Molecular Genetics University of California Davis CA USA; ^4^ Western Institute for Food Safety and Security School of Veterinary Medicine University of California Davis CA USA; ^5^ Genome Center University of California Davis CA USA

**Keywords:** avian influenza virus detection, M‐RTPCR, poultry industry, ultrafiltration, waterfowl, wetlands

## Abstract

Migratory waterfowl, including geese and ducks, are indicated as the primary reservoir of avian influenza viruses (AIv) which can be subsequently spread to commercial poultry. The US Department of Agriculture's (USDA) surveillance efforts of waterfowl for AIv have been largely discontinued in the contiguous United States. Consequently, the use of technologies to identify areas of high waterfowl density and detect the presence of AIv in habitat such as wetlands has become imperative. Here we identified two high waterfowl density areas in California using processed NEXt generation RADar (NEXRAD) and collected water samples to test the efficacy of two tangential flow ultrafiltration methods and two nucleic acid based AIv detection assays. Whole‐segment amplification and long‐read sequencing yielded more positive samples than standard M‐segment qPCR methods (57.6% versus 3.0%, *p* < .0001). We determined that this difference in positivity was due to mismatches in published primers to our samples and that these mismatches would result in failing to detect in the vast majority of currently sequenced AIv genomes in public databases. The whole segment sequences were subsequently used to provide subtype and potential host information of the AIv environmental reservoir. There was no statistically significant difference in sequencing reads recovered from the Rexeed^TM^ filtration compared to the unfiltered surface water. This overall approach combining remote sensing, filtration and sequencing provides a novel and potentially more effective, surveillance approach for AIv.

## 1 INTRODUCTION

Wild birds of the *Anseriformes* and *Charadriiformes* order represent a natural and asymptomatic reservoir for avian influenza viruses (AIv) that infect domestic, captive and wild free‐ranging bird species (Pantin‐Jackwood & Swayne, 2009). Due to the presence of influenza A viruses in waterfowl which can be excreted from faecal/oral routes into the environment (Ronnqvist, Ziegler, von Bonsdorff, & Maunula, 2012), surveillance of AIv's in waterfowl habitat play a vital role in the transmission of AIv (Ito et al., 1995; Keeler, Berghaus, & Stallknecht, 2012; Lang, Kelly, & Runstadler, 2008; Markwell & Shortridge, 1982). While current national surveillance of AIv in commercial and backyard poultry is rather extensive temporally and spatially, the source waterfowl population, remains relatively under‐surveilled. Specifically, in 2018 the USDA discontinued the interagency HPAI Wild Bird Early Detection System and currently only a minimal level of active surveillance in Alaska and a few isolated regions is being implemented (Liberto, 2019).

When wetland habitat maintains the optimal conditions of low temperatures (<17°C), slightly basic pH (7.4–8.2), and low salinity (0–20,000 parts per million (ppm)), the potential for AIv to persist and remain infectious in the environment exists (Brown, Goekjian, Poulson, Valeika, & Stallknecht, 2009). Specifically, the faecal/oral excretion of AIv into the environment leads to heavy contamination and seeds the pathway to indirect transmission of AIv to susceptible birds from water and sediment (Lang et al., 2008; Nazir, Haumacher, Ike, & Marschang, 2011). Surveillance efforts in aquatic environments are necessary to understand the environmental persistence of AIv (Pepin et al., 2019), but sampling efforts must consider complex factors when analysing natural water samples such as accessibility to large volumes of water and maintaining conditions of water (e.g. pH, temperature) to ensure virus is not degraded in transport (Keeler et al., 2012). Viral particles within aquatic environments are thought to set the patterns of transmission within waterfowl (Roche et al., 2009) indicating a need for high surveillance of water and sediment of these wetland habitats (Ronnqvist et al., 2012). Detection of virus in these aquatic environments could offer a complementary surveillance approach and provide a novel predictive level of AIv molecular ecology (Pepin et al., 2019).

However, current detection methods for AIv in water lack sensitivity, are very limited, and are not representative of AIv ecology within whole habitats (Stallknecht, Goekjian, Wilcox, Poulson, & Brown, 2010). Specifically, detection of AIv in wetlands is typically done via the collection and PCR based analysis of multiple small (~1 ml or less) surface water samples with no concentration methods (Henaux, Samuel, Dusek, Fleskes, & Ip, 2012). For example, to quantify the prevalence of AIv in California's Central Valley wetlands, Henaux et al. (2012) collected a total of 597 surface water samples and performed RNA extractions on a 50 μl aliquot from each 45 ml sample (Henaux et al., 2012). LPAI was detected in 2% of their samples by matrix gene real time Reverse Transcription‐Polymerase Chain Reaction (RT‐qPCR) (Spackman et al., 2002) and no virus was isolated from surface water samples (Henaux et al., 2012). Although this experimental design yields a higher sample size, small volume samples may not be representative of the entire wetland ecosystem.

Combining a more sensitive environmental AIv sampling technique with more targeted sampling of wetlands where waterfowl occur in high densities could lead to greater efficiency and effectiveness of surveillance. NEXt generation RADar (NEXRAD) is a remote sensing tool that offers the ability to quantify waterfowl density and distribution (Buler et al., 2012). Specifically, NEXRAD provides an instantaneous measure of radar reflectivity at the onset of highly synchronized flights of waterfowl departing their daytime roosting locations as they fly to their night‐time feeding locations (Buler et al., 2012).

The goal of this study was to develop a sensitive and targeted detection method for AIv in wetlands with high waterfowl density as a foundational step to improving environmental surveillance. To reach this goal, we: (a) used NEXRAD observations to identify two wetlands with high waterfowl density, (b) tested two filtration methods to concentrate AIv in water samples from those wetlands, (c) tested two nucleic acid detection methods (e.g. Whole‐segment amplification and long‐read sequencing versus matrix segment RT‐qPCR), and (d) provided sequence information detailing the molecular viral ecology of AIV in sampled wetlands.

## 2 MATERIALS AND METHODS

### 2.1 NEXRAD wetland selection

**Figure 1:**
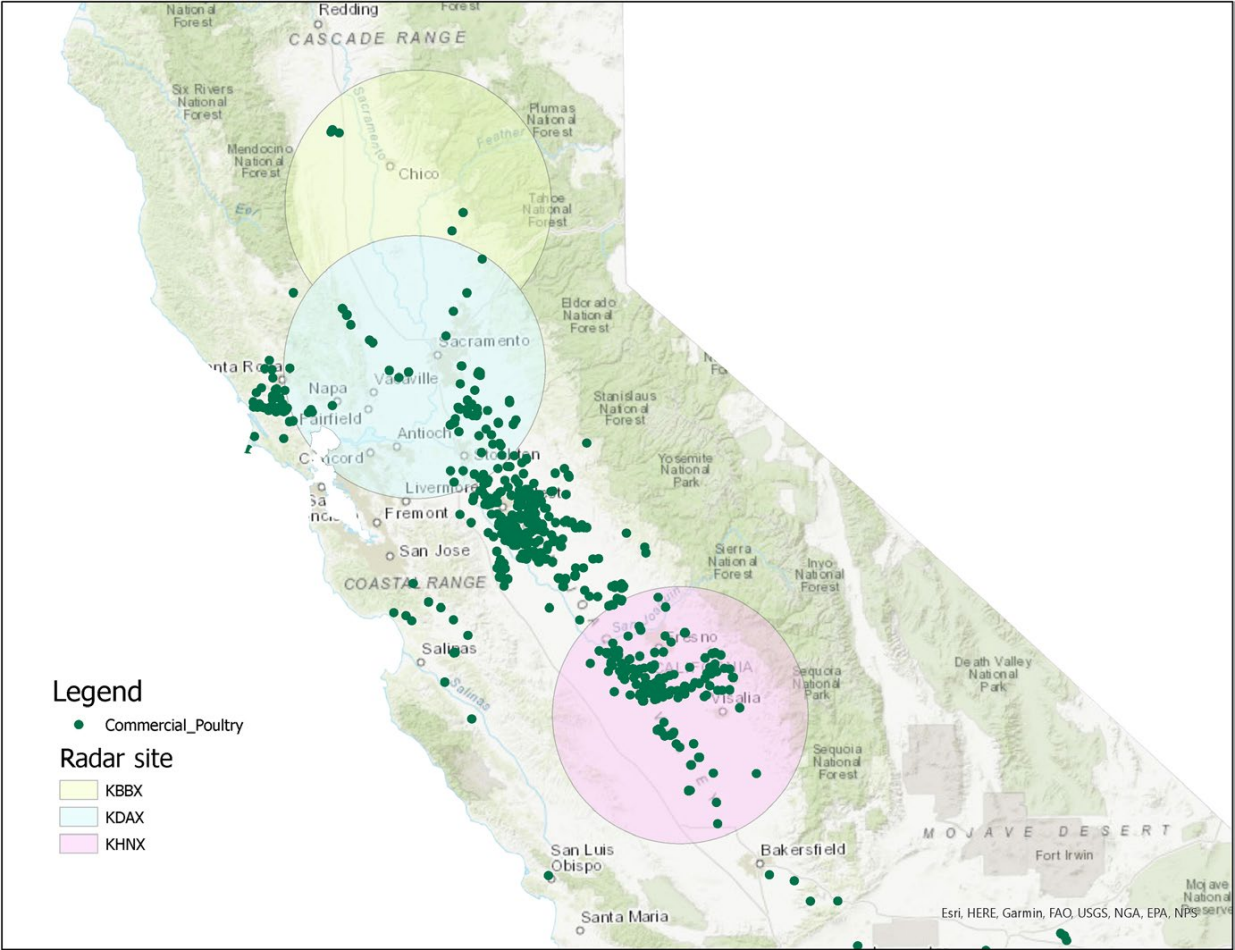
Location of NEXRAD radar stations (100 km radius coverage areas), KBBX, KDAX, KHNX, in relation to California poultry facilities

Historic (i.e. 2014) radar reflectivity from three NEXt generation RADar (NEXRAD) stations (KBBX, KDAX and KHNX) in the Central Valley of California were used to identify wetlands with high waterfowl density and distribution (Figure 1). NEXRAD is a remote sensing tool proven to quantify waterfowl density and distribution near the ground using an instantaneous measure of radar reflectivity at the onset of highly synchronized flights of waterfowl departing their daytime roosting locations as they fly to night‐time feeding locations (Buler et al., 2012).

Data between 7.5 and 100 km range from the radar were considered for analysis. For each sampling night free of precipitation and anomalous propagation of the radar beam, we interpolated reflectivity measurements to the instant when the onset of bird flight reaches its peak rate of increase (i.e. typically near the end of evening civil twilight) and estimated the vertically integrated reflectivity from 0‐2 km above the ground for each sample volume following (Buler et al., 2012). This approach produces a continuous surface map of the relative density of birds aloft across the radar domain at the peak of flight exodus to maximize the spatial correlation with their diurnal ground roosting density.

The Yolo Bypass Wildlife Area in Yolo County and a private hunting club in Butte County were selected as representative wetland habitats. The wetland in Butte County operates as a private hunting club with adjacent rice and agricultural fields. Yolo Bypass Wildlife Area is public land with private agricultural lands surrounding it. Permission to collect water from each wetland was obtained by land managers prior to sampling. Both locations are used for seasonal hunting due to the abundance of birds that utilize the habitat throughout fall and winter.

### 2.2 Sample collection

**Figure 2:**
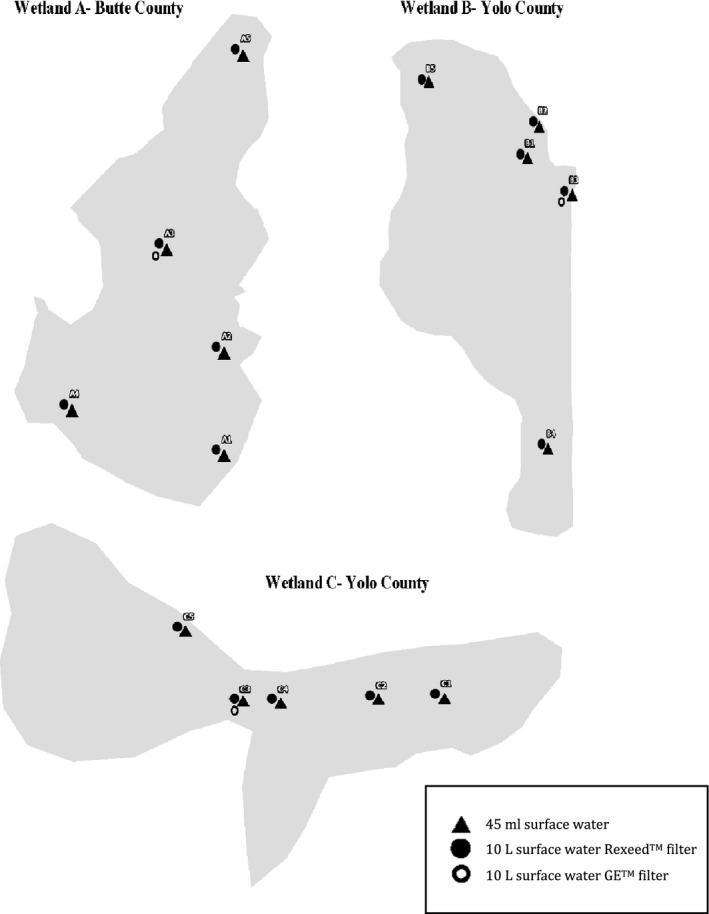
Radar‐observed mean daily waterfowl density from November 2014 to February 2015 in conjunction with sampling locations in Colusa County and Yolo County, California, USA

Water samples were collected between June 2018 and September 2018. During each sampling interval, five locations were chosen randomly with GPS marking. Samples were collected between the surface and approximately 1m of depth. Measurements of pH, temperature, and salinity were recorded with the YSI Professional Plus sensor at each of the five locations within a wetland. Due to equipment error, the pH, temperature salinity were not recorded at the first sampling interval in Butte County. At each of the five locations, a 10‐litre water sample was collected according to the lower limit of large volumes considered to be adequate for determining pathogen presence in water (Morales‐Morales et al., 2003). At the third sampling location within a wetland, a second 10‐L carboy was collected for a total of six 10‐litre carboys to be filtered with ultrafiltration (Figure 2). A single 45 ml surface water sample was collected from each of the five locations to compare with previous sampling methods. A total of five 45 ml sediment samples were collected at each wetland interval to compare the presence and persistence of AIv in sediment to water samples (Nazir et al., 2011). Water samples were stored on ice and taken back to the lab for same day filtration.

### 2.3 Tangential flow ultrafiltration

**Figure 3:**
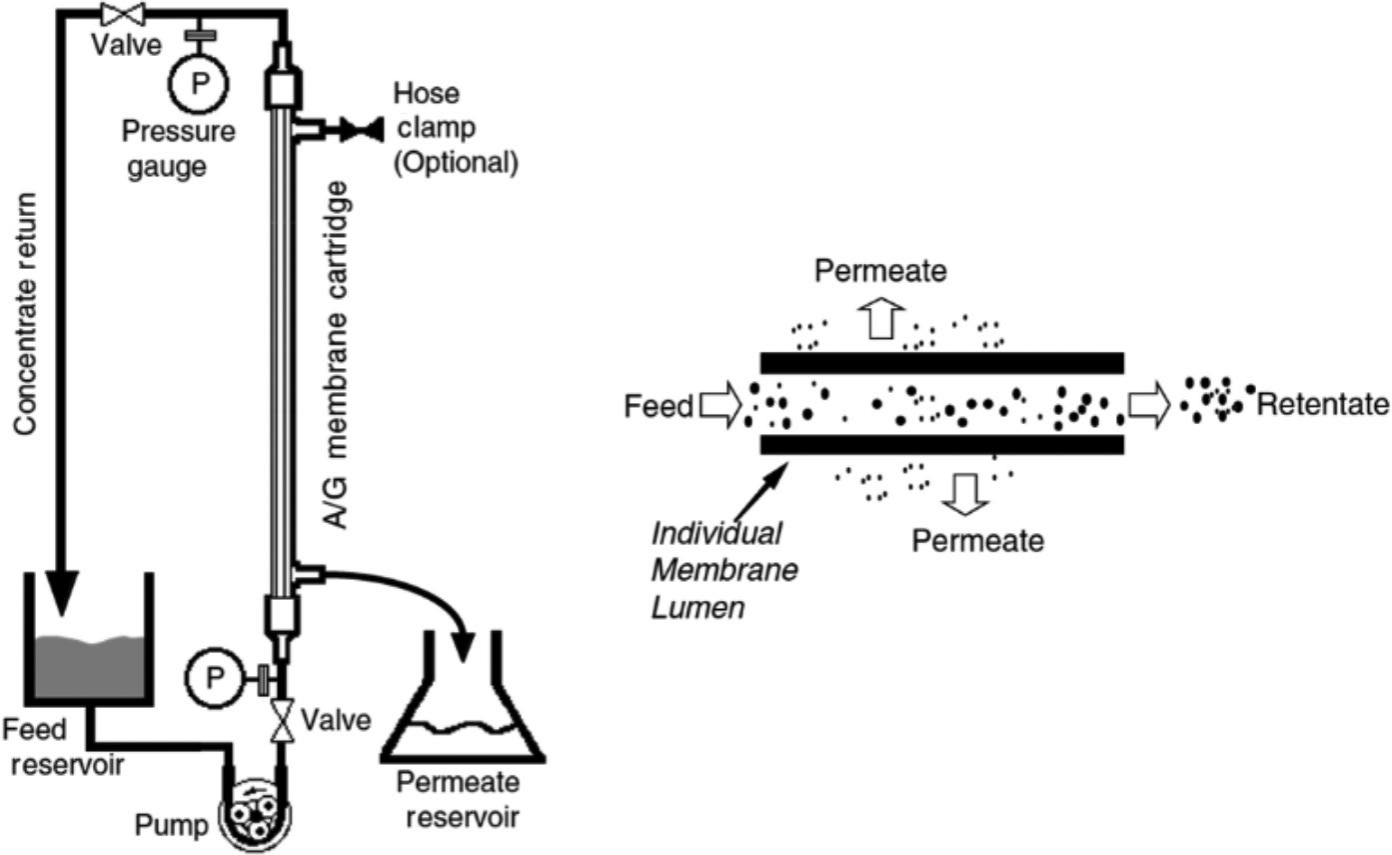
As water is fed through the membrane the lumen retains the viral particles circulating within the system. Simplified tangential flow schematic extracted from GE’s Hollow Fiber Filter Cartridge's Operating Handbook

The rationale of ultrafiltration is to concentrate large volumes of wetland water for a more representative sample that is indicative of overall AIv presence in the environment. Conventional tangential flow ultrafiltration separates solutes that differ by tenfold in size through membrane pore size, qualifying this method of filtration as an appropriate approach for AIv detection in larger volumes of water (Figure 3) (Christy, Adams, Kuriyel, Bolton, & Seilly, 2002). Viral particles were retained by molecular weight cut‐offs and concentrated in the retentate while molecules smaller than the filter's pore size flowed through the membrane (Figure 3) (Hill et al., 2005).

The single use Asahi Kasei Rexeed^TM^ 25s filter with a 30 kDa molecular weight cut‐off and membrane area of 2.5 m^2^ was compared with the GE^TM^ UFP‐3‐C‐4X2MA autoclavable column with a 3 kDa molecular cut‐off and membrane area of 0.14 m^2^ (Partyka, Bond, Chase, Kiger, & Atwill, 2016) (Figure 3). Prior to filtration, each filter was primed with 1litre of blocking solution of NaPP and deionized water. Five of the 10‐litre carboys were filtered using individual Asahi Kasei Rexeed^TM^ 25s columns, and the sixth 10‐litre carboy collected at the third sampling interval was filtered using the GE^TM^ UFP‐3‐C‐4X2MA column. Each 10‐litre carboy was filtered down to a 45 ml retentate to be comparable with the 45 ml unfiltered surface water sample. Flow rates were observed based on each of the manufacture's recommendations. Pressure of the filtration system did not exceed 20 psi. Upon completion, each filter was eluted with a 500 ml solution of NaPP, Tween, Antifoam, and deionized water.

### 2.4 PCR and sequencing

RNA from water and soil samples were extracted using the QIAamp Viral RNA Mini Kit (QIAgen) on a QIAcube and the PowerViral Environmental DNA/RNA Isolation kit (QIAgen), respectively. Following extraction, two methods were used to detect the presence of AIv in samples: reverse‐transcriptase quantitative polymerase chain reaction (RT‐qPCR) and whole segment amplification followed by sequencing. With respect to the RT‐qPCR, a conserved ~100 bp fragment of the matrix protein is amplified according to Spackman et al. (2003). RT‐qPCR was performed using this method in order to determine the limit of detection (Figure S1). We reference this method as RT‐qPCR hereafter.

Whole‐segment amplification was attempted using multi‐segment RT‐PCR (Zhou et al., 2009). This procedure uses primers that are complementary to genome segment packaging regions (uni12 and uni13), which are conserved among all influenza A viruses, including AIv. Thus, this procedure amplifies entire gene segments if they are present in the sample. We conducted gel electrophoresis to assess genome segment amplification and completed multiplexed sequencing of amplicons using the Oxford Nanopore MinION sequencer (Oxford Nanopore Technologies). The primers included overhangs with 5′ 22 bp barcodes (shared among all samples) and 3′ 8 bp barcodes that were unique for each sample. The MinION sequenced single DNA molecules and allowed for the recovery of entire influenza genome segments (Imai et al., 2018; Wang, Moore, Deng, Eccles, & Hall, 2015). We reference this method as amplification/sequencing hereafter.

### 2.5 Bioinformatics analyses

Output from the MinION sequencer was analysed using a custom pipeline that is openly available online. Briefly, raw signal files (.fast5 format) were base‐called using Guppy in high accuracy base calling mode (HAC). After quality filtering using Nanofilt (De Coster, D’Hert, Schultz, Cruts, & Van Broeckhoven, 2018), reads were demultiplexed (i.e. assigned to a sample) and primers trimmed using cutadapt (Martin, 2011) with exact matches for sample‐identifying barcodes. We used a single brand‐new flow cell and included negative and positive controls throughout the sample workflow. NCBI command line BLAST using GNU Parallel (Tange, 2011) was used to search demultiplexed files against all avian influenza whole genome sequences available in the NIAID Influenza Research Database (IRD) (Zhang et al., 2016). Sample metadata from IRD was used to annotate likely subtypes and hosts of AIv sequences detected in each collecting location, based on the closest match in the IRD.

### 2.6 In‐silico evaluation of M segment RT‐qPCR

To evaluate the efficiency of the specific M protein RT‐qPCR procedure we used herein (Spackman et al., 2003), we conducted two computational analyses on all fully sequenced avian influenza genomes in IRD as of August 12, 2019. First, we searched for exact matches for primers and probes and used this as an initial indicator of the probability of a successful assay. Second, to conduct a more realistic analysis of assay success (as PCR often tolerates primer mismatches), we conducted a thermodynamic simulation of the TaqMan assay using ThermonucleotideBLAST (Gans & Wolinsky, 2008) which outputs whether an amplicon was generated (together with its length and sequence) for each sequence under the specified conditions.

To verify discrepancies between positive samples detected with RT‐qPCR versus amplification/sequencing, we employed the same two analyses to examine results for our field‐collected samples.

### 2.7 Statistical analysis

To compare the efficiency of RT‐qPCR with amplification/sequencing in detecting AIv from samples, we calculated the number of positive samples under each method and compared them using a proportion test. To test whether filtration methods increased the proportion of AIv positive samples or the number of AIv sequences detected in samples, we conducted a proportion *t*‐test for each filtration method. All statistical analyses were conducted using R. Code and data are available online at https://github.com/sociovirology/aiv_detection_environment.

## 3 RESULTS

### 3.1 NEXRAD data

**Figure 4:**
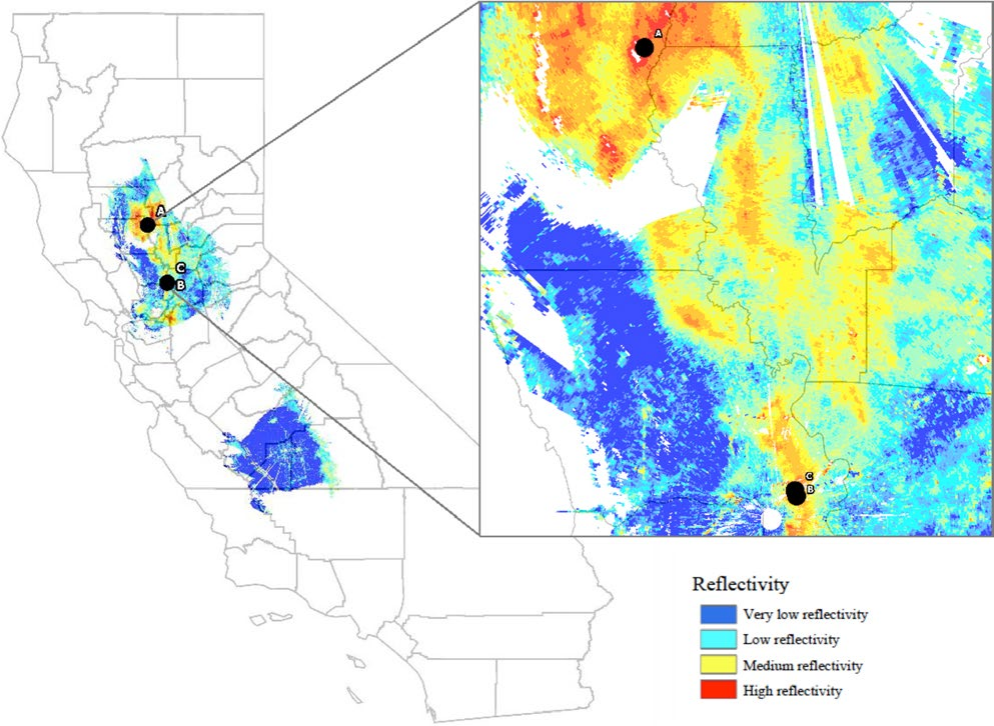
Radar‐observed mean daily waterfowl density from November 2014 to February 2015 in conjunction with sampling locations in Colusa County and Yolo County, California, USA

The NEXRAD generated surface map allowed for the selection of wetland environments where we would expect to see viral presence due to the waterfowl population density. Using the NEXRAD data collected and analysed, two watersheds in the California Central Valley were selected as our study sites (Figure 4).

### 3.2 Water quality measurements aligned with stability threshold

Water quality variables taken from sample intervals B and C indicated that pH and temperature was at the upper limit of the thresholds while salinity was within lower range of the threshold (Table 1). Previous studies suggest that virus stability can be observed between neutral and pH of 8.5, lower temperatures around 17°C and low saline conditions (Brown et al., 2009; Stallknecht, Shane, Kearney, & Zwank, 1990). The pH and temperature conditions we measured within our Yolo County wetlands were within the upper limits stated for viability of AIv.

**Table 1:** Temperature, salinity and pH measurements from Butte County wetland (A1‐5), Yolo Bypass Wildlife Area in July 2018 (B1‐5) and Yolo Bypass Wildlife Area in September 2018 (C1‐C5)

Date	6/25/2018					7/31/2018					9/8/2018					AVG	Median
Location	A1	A2	A3	A4	A5	B1	B2	B3	B4	B5	C1	C2	C3	C4	C5		
Temperature (ºC)	–	–	–	–	–	18.3	18.9	20.5	21.3	20.1	17.6	16.8	19.8	17.1	18.8	18.9	18.9
Salinity (ppm)	–	–	–	–	–	375	414	435	249	392	794	791	771	784	777	578	603
pH	–	–	–	–	–	9.37	9.02	8.97	7.73	8.79	8.34	8.33	8.18	8.24	8.13	8.51	8.34

### 3.3 Recovery of avian influenza sequences from water samples with and without filtration

**Figure 5:**
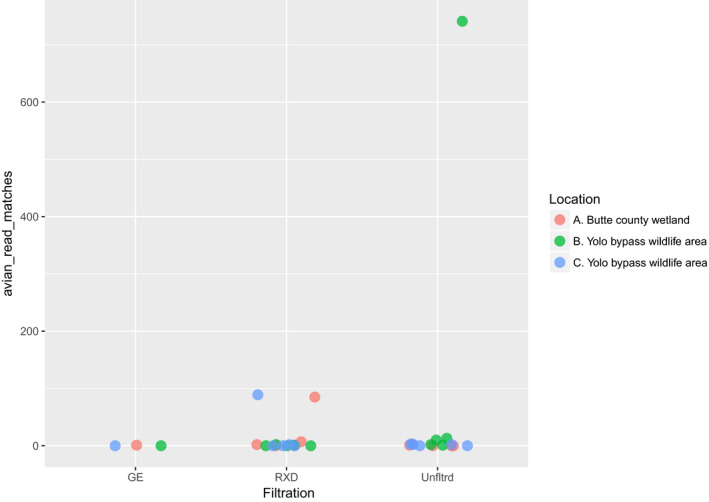
Frequency of filtered and unfiltered positive reads with an outlier from sample interval B. at Yolo Bypass Wildlife Area. GE = GE^TM^ UFP‐3‐C‐4X2MA RXD = Asahi Kasei Rexeed^TM^ 25s. Unfltrd = 45 ml unfiltered surface water

**Figure 6:**
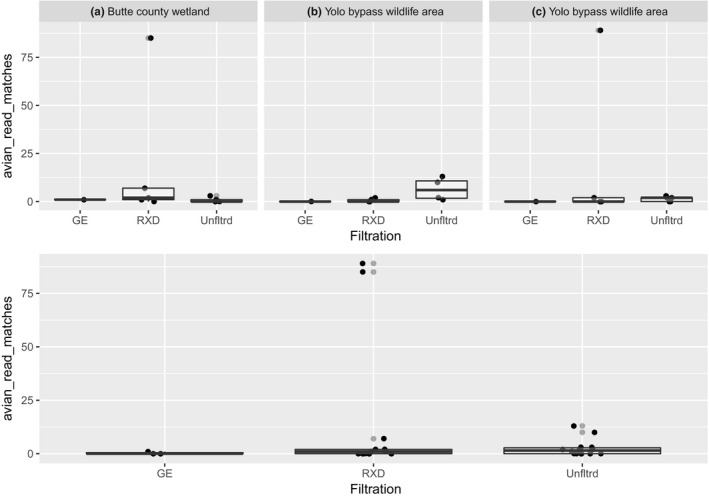
Rexeed^TM^ filtration yields more AIv sequencing reads than unfiltered samples (excluding outlier). Filtration techniques with number of positive reads for AIv. GE = GE^TM^ UFP‐3‐C‐4X2MA RXD = Asahi Kasei Rexeed^TM^ 25s. Unfltrd = 45 ml unfiltered surface water. Top panels show data by site, bottom is in aggregate. These panels exclude an outlier sample that yielded > 700 AIv reads

We did not detect influenza virus in sediment samples thus, we focus on water samples hereafter. We compared two filtration methods to unfiltered water: GE^TM^ UFP‐3‐C‐4X2MA and Asahi Kasei Rexeed^TM^ 25s. We recovered only a single positive sample with one read using GE filtration (*n* = 3). The proportion of samples that were AIv positive under Rexeed filtration (0.533) versus. unfiltered surface water (0.667) was not statistically significantly different (X^2^ = 0.13889, *df* = 1, *p* = .7094, 95% CI: −0.547 to –0.281). Among those samples that were positive, unfiltered surface water yielded the more AIv‐matching reads on average (77.8 ± 223, *n* = 10), compared to Rexeed filtration yielded more AIv reads (23.6 ± 39.2, *n* = 8). However, this average was heavily weighted by one unfiltered water sample from the Yolo Bypass area, which had over an order of magnitude more reads than any other unfiltered sample (Figure 5). We removed this outlier sample and while average reads from Rexeed filtration were higher on average (23.6 ± 39.2, *n* = 8) than unfiltered surface water (4.11 ± 4.31, *n* = 9), a *t*‐test indicated this difference wasn't statistically significant (t = 1.401, *df* = 7.151, *p* = .203) (Figure 6).

### 3.4 Whole‐segment amplification/sequencing yielded more positive samples than M‐segment RT‐qPCR

RT‐qPCR (as implemented in Spackman et al., 2003) yielded a single positive sample out of 33 tested samples (excluding five sediment samples which were negative). Amplification/sequencing yielded AIv detection (defined as ≥1 read matching AIv database) in 19/33 samples. If positives by amplification/sequencing were restricted to ≥1 read matching only the M‐segment (which is the target of the RT‐qPCR), the number of positive detections would then be 15/33 samples. The difference in the proportion of positive samples using RT‐qPCR (3.0%) versus amplification/sequencing (57.6%) was statistically significant (X^2^ = 20.733, *df* = 1, *p* = 5.281e‐06, 95% CI: −0.754 to –0.337).

To examine the discrepancy in positive sample detection between these two approaches, we used the sample sequence data we obtained to search for matches to the primers. The 24 nucleotide primers and probe as published in Spackman et al. (2003) did not get any exact matches. Since the first 3’ bases are the ones that are most critical for annealing, we conducted an exact match search for the first 16nt of the primers and probe and obtained 61 matches in sample B1_SW_Unfltrd, the only RT‐qPCR positive sample we found. A computational thermodynamic simulation of the Taqman assay using published conditions predicted no amplification in any of the samples. However, at relaxed annealing temperature of 50º C (compared to published 60ºC) 84 molecules predicted to amplify the target in for the same sample that indeed was positive in the lab: B1_SW_Unfltrd.

To examine whether this discrepancy was particular to our samples, we conducted a similar primer search on all M‐segment sequences (*n* = 14,402) available from all complete AIV genomes in the IRD as of Aug 12, 2019. This analysis suggests an overall success rate of 232/9855 = 0.02354135 ~2.35%, based on exact primer matches to the full‐length primers published in Spackman et al. (2003). Based on exact matches to the first 3′ 16nt of the primers/probe, the overall success rate increases to 51.16%. If exact matches are required for amplification, at best, half of known fully sequenced avian influenza viruses would be detectable. However, the success rate could be higher given that PCR is able to tolerate mismatches. A thermodynamic assay simulation suggests no amplification based on published PCR conditions (60ºC). Under more permissive conditions (50ºC annealing), the analysis suggests an overall success rate of 52.20% and at the very low 40ºC annealing temperature 91.83% of samples should generate positives under the TaqMan assay.

### 3.5 Sequencing data and AIv database match summary

The R 9.4.1 MinION flow cell yielded 222,316 total reads, of which 143,716 passed quality control (q > 9). After removing non‐target sequences (achieved through trimming of known primer sequences), we obtained a total of 47,962 reads, of which 4,782 matches to all sequenced AIv genomes in the IRD. Samples that yielded electrophoretic bands corresponded well with the number of sequencing reads obtained, providing further evidence confirming the specificity of our primers (Figure S2). We verified that the pipeline correctly assigned reads by (a) monitoring that multiple negative controls (*n* = 4) obtained no reads and by (b) aligning reads from the positive control to its reference genome, of which >92% aligned to the reference (see code for details). No sequences were identical within or across samples. We excluded reads from positive controls and hereafter concentrate on 968 reads. For our sample reads (*n* = 968) the AIv matches were at an average of 91.64% (± 3.61) identity with an average query coverage of 1,217.43 base pairs (± 506.66). Thus, for many of our reads we were matching the entire lengths of segments in the database with high identity. For instance, for the longest influenza segment (PB1 = 2,341 bp) we had 71 sequences >2,300 base pairs with an average of 90.83% identity. The majority of segments matched in the database (Table 2) were M1/2 (segment 7 = 392) and NA (segment 6 = 395 reads). An additional 4 segments (PB2, PA, HA, NS1) had at least >10 database matches. We found no matches to PB2 (segment 1) and NP (segment 5).

**Table 2:** Number of reads amplified and sequenced from water that matched a database of all avian influenza virus whole genomes in the NIAID Influenza Research Database (IRD)

Segment number	Segment name	Reads matching AIV database (IRD)
1	PB2	0
2	PB1	87
3	PA	14
4	HA	59
5	NP	0
6	NA	395
7	M	392
8	NS	21

### 3.6 California Wetlands harbour avian viruses from multiple potential host origins and subtypes

**Figure 7:**
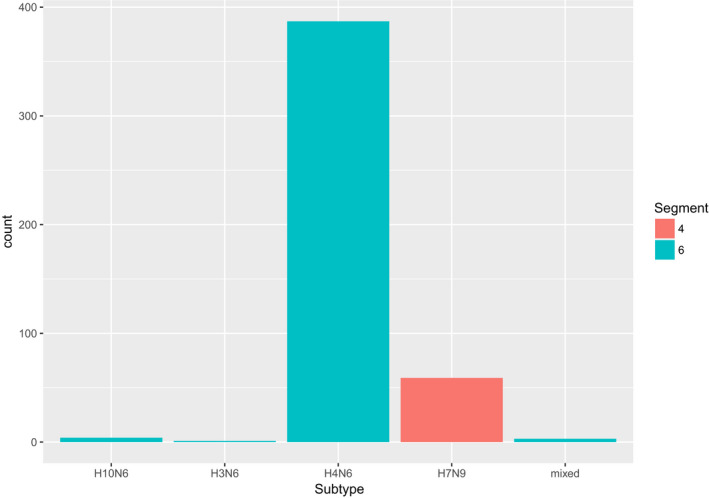
H7 and N6 are the most prevalent subtypes recovered from sequencing reads. The x axis shows the subtype of the nearest AIv match in the database. Subtype matches are colored by the segment corresponding to the sequencing read recovered: segment 4 (HA) and segment 6 (NA).

We determined the likely subtype of HA and NA segments (*n* = 454) based on the annotated subtype of the genome segment matched in the database (Figure 7). We found that N6 (*n* = 392) and H7 (*n* = 59) composed all the confirmed subtype matches; 3 NA matches were annotated in IRD as ‘mixed’ subtype.

**Figure 8:**
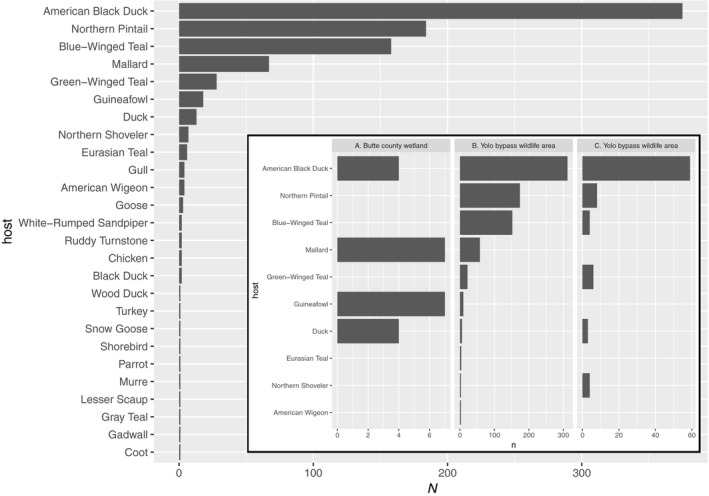
Potential hosts for AIv sequences recovered by location (inset). The y axis denotes the number of sequences from our study that matched a genome associated with a particular host in a database including all the AIv in FluDB. Positive reads that match one segment in >500 bp from one fully sequenced viral genome in FluDB were kept for analysis. Filtered and unfiltered samples were pooled for site breakdown.

In order to understand potential avian hosts of specific AIv's, we examined the host of the virus in the IRD that our sequences matched to gain insight into the potential hosts of the AIv sequences found in samples (Figure 8). Among the database matches that were >500 bp (*n* = 885), there were 26 avian hosts. Five of these hosts accounted for 91.75% of matches: American Black Duck (*Anas rubripes*) 42.37%, Northern Pintail (*Anas acuta*) 20.79%, Blue‐winged teal (*Anas discors*) 17.85%, Mallard (*Anas platyrhynchos*) 7.57%, and Green‐Winged Teal (*Anas carolinensis*) 3.16%.

## 4 DISCUSSION

The results of this study provide evidence for the feasibility of an AIv monitoring approach that combines various methods and technologies with respect to waterfowl surveillance and AIv detection. Specifically, the ability to remotely identify targeted wetlands for wetland water sampling (filtered versus. unfiltered) linked to PCR based AIv analyses (matrix segment RT‐qPCR versus whole‐segment amplification/sequencing) is a unique approach that should be considered for AIv surveillance.

Initial tests suggested AIv sequence recovery was not different in filtered samples compared to unfiltered surface water. However, our data set included an unfiltered sample that yielded over an order of magnitude more sequencing reads than any other sample. Excluding this outlier sample, filtration of environmental water samples by Asahi Kasei Rexeed^TM^ 25s yielded more sequence data, on average, compared to unfiltered surface water samples, presumably by retaining more viral particles. However, this difference was not statistically significant, likely due to the small number of samples. The outlier sample possibly represents a ‘jackpot’ scenario of sampling an area of high AIv. While more sampling is needed to better establish the effect of filtration on viral recovery over multiple seasons, the results point to the potential efficacy of targeted wetland surveillance without filtration. The two filters likely performed differently due to the molecular weight cut‐off (MWCO) and surface area/flow rate differences of the two filters. The larger pore size and overall design of the Asahi Kasei Rexeed^TM^ 25s was proven to be better fit at retaining viral particles.

While filtration did not improve read recovery, the whole‐segment amplification/sequencing approach yielded sensitive detection from unfiltered surface water samples that were missed using the standard, published M‐segment RT‐qPCR approach (Spackman et al., 2003). Regardless of filtration method, RT‐qPCR yielded one positive sample (out of 33 or 3.0%), versus 19 samples (57.6%) using sequencing. Thus, this amplification/sequencing approach could be a powerful alternative to RT‐qPCR, whether filtration is used or not. While the comparison is not completely appropriate due to differences in sample location and time, a previous efforts using RT‐qPCR in California wetlands detected LPAI in 2% of the 597 samples collected (Henaux et al., 2012).

With respect to the RT‐qPCR and amplification/sequencing methods it is important to note that these methods have different goals by design. The RT‐qPCR detects a 100bp fragment of a particular influenza segment (M1/M2 matrix gene). The MinION sequencing platform, as used here, can sequence full influenza genome segments relying on the conservation of packaging regions (Zhou et al., 2009). The results herein, together with the known molecular instability of RNA in solution, potentially suggest that we were recovering RNA directly from the capsids, which would protect RNA from damage or degradation prior to RNA extraction. It is extremely unlikely to obtain sequencing reads >2.2 kpbs (*n* = 80) of RNA with even low levels of RNA degradation. This is in contrast to RT‐qPCR, which by design only amplifies a small conserved segment of RNA, that may or may not be representative of intact viral particles present. Further testing including electron microscopy and virus isolation is needed to determine to what extent sequences are in fact derived from intact viral particles, and whether those particles retain infectivity.

An additional advantage of the amplification/sequencing approach is that it permits the sequence data to be used to produce a more detailed characterization of the AIv environmental reservoir. For instance, we were able to determine likely subtypes of HA and NA segments (Figure 7) without additional tests. However, it is important to understand the nature of the sequencing data and its limitations for drawing conclusions. This approach sequences entire genome segments, but the linkage between these segments (i.e. which segments occupied the same capsid) is lost during RNA amplification. This means that inferences from database annotations for subtype cannot use the M‐segment, but must be restricted only to HA and NA segments as we have done here. We also used available data in FluDB to gather a list of potential hosts for the AIv sequences we characterized (Figure 8). We note that many of these host species are present in these California wetlands and correspond to typical reservoir species for AIv. It is important to emphasize that many AIv's can have a wide host range among avian and non‐avian species, so these are just potential hosts. To that point, the most prevalent species noted in Figure 8 is the American Black Duck, which is not present in California. The American Black Duck is closely related to the Mallard (very abundant in CA), so one potential possibility is that many of the same AIV types have adapted to these two species. In addition, from a sequencing perspective, it is worth noting that the per‐base sequencing accuracy of MinION sequencing is lower than other sequencing approaches. In particular, database matches can be affected by the MinION error rate (empirically calculated using a positive control to be ~15%), in turn affecting subtype assignments or potential hosts as reported herein. Thus, care must be taken in drawing conclusions that rely crucially on single nucleotide genotypes. However, this is also the case with other sequencing approaches with higher per‐base accuracy rates. For instance, Illumina sequencing delivers high per‐base accuracy, but genotyping from this type of environmental sample is contraindicated, because it requires the assumption that sequences came from the same molecule, when it is almost certain they do not because cDNA is fragmented for sequencing. In contrast, with Nanopore MinION it is almost certain those bases came from the same molecule, which is more important for environmental detection of AIv as recovery of whole genome segments from influenza suggest that they are derived from virions. Furthermore, new approaches bring MinION sequencing per‐base accuracy up to Illumina levels (Karst et al. 2020). In sum, the relevance of the advantages and disadvantages of any given detection method depend on the research goals and it is important to have an ample toolkit for AIv surveillance.

One potential implication of the amplification/sequencing results compared to the RT‐qPCR results is that AIv prevalence in the wetlands is higher than previously supposed (Henaux et al., 2012) and hence the role of wetlands in seeding new infections may also be larger than previously supposed. While we did not do virus isolation in order to confirm infectivity, the higher prevalence of positives in amplification/sequencing compared to RT‐qPCR in addition to our pH, temperature and salinity data (Table 1) suggests that conditions for infectivity of the viruses are largely met (Brown et al., 2009). This would support our current understanding of one route of transmission where the excretion of infected faeces in the environment leads to ingestion from susceptible birds completing the faecal environmental transmission route (Breban, Drake, Stallknecht, & Rohani, 2009).

The needs for sensitive methods of AIv detection are in high demand (Deboosere et al., 2011; Henaux et al., 2012; Keeler et al., 2012; Khalenkov, Laver, & Webster, 2008; Ronnqvist et al., 2012; Stallknecht et al., 2010; Zhang, Li, Chen, Chen, & Chen, 2014). This sequence data provides information on the genetic diversity and composition of influenza viruses in the water column. Sequence data could be used to determine influenza virus subtypes present, infer time‐space influenza virus sequence dynamics, and relate these sequence patterns to larger scale, ongoing influenza virus dynamics using independent surveillance data. Furthermore, assessing the strength of the link between remotely sensed waterfowl density and viral load in the environment is needed. This could be done by testing spatio‐temporal correlations of AI prevalence in environmental samples with concurrent radar‐observed bird density at multiple sampling locations repeated over short time intervals (e.g., bi‐weekly or monthly). Such analyses will enhance our knowledge on the nature of the AIv waterfowl reservoir and allow us to couple remotely sensed patterns of bird movements to the risk of specific AIv groups for a strong surveillance tool.

## CONFLICT OF INTEREST

The authors declare no conflict of interest.

## ETHICAL STATEMENT

No samples were collected from animals and no surveys were gathered from human subjects for this study.

## Supporting information

Figures S1 and S2

## Data Availability

The data and code that support the findings of this study are openly available. Sequence data was deposited in the NCBI Sequence Read Archive (Accession SRX7014890) and is available at https://www.ncbi.nlm.nih.gov/sra/SRX7014890. Data and code for analysis is available on GitHub https://github.com/sociovirology/aiv_detection_environment
