# Supplementary figures and images for: A comparison of amplification methods to detect Avian Influenza viruses in California wetlands targeted via remote sensing of waterfowl
Source: Transbound Emerg Dis. 2020 Jun 27;68(1):98–109. doi: 10.1111/tbed.13612 (PMC8048853; doi:10.1111/tbed.13612)

## Slide 1
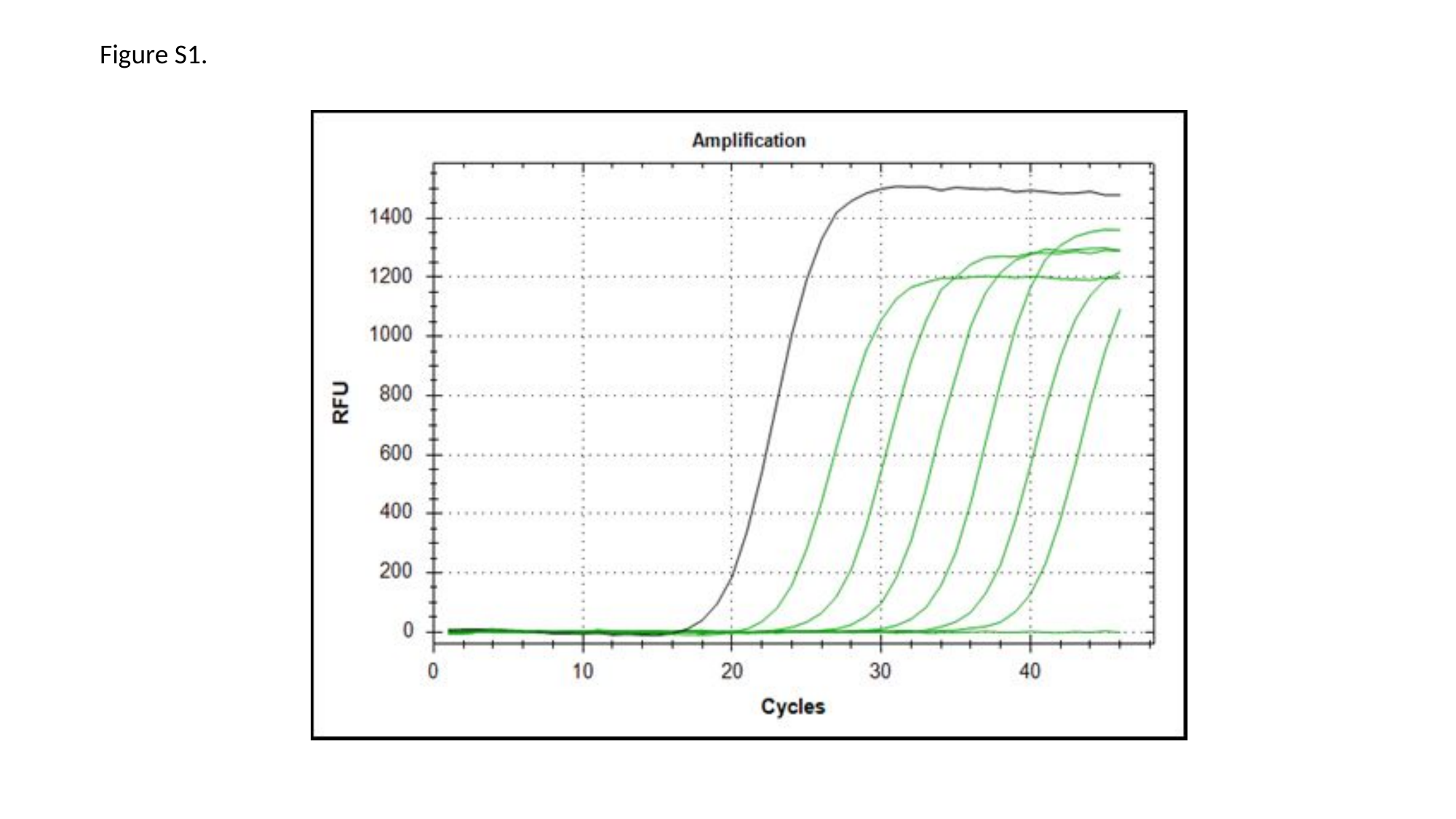

Figure S1.

Supplement: Supplementary file 1 — Figures S1 and S2 [file TBED-68-98-s001.pptx]
